# Droplet digital PCR analysis of *NOTCH1* gene mutations in chronic lymphocytic leukemia

**DOI:** 10.18632/oncotarget.13246

**Published:** 2016-11-09

**Authors:** Angela Minervini, Francesco Crescenzio Minervini, Luisa Anelli, Antonella Zagaria, Paola Casieri, Nicoletta Coccaro, Cosimo Cumbo, Giuseppina Tota, Luciana Impera, Paola Orsini, Claudia Brunetti, Annamaria Giordano, Giorgina Specchia, Francesco Albano

**Affiliations:** ^1^ Department of Emergency and Organ Transplantation (D.E.T.O.), Hematology Section, University of Bari, Bari, 70124 Italy

**Keywords:** NOTCH1, chronic lymphocytic leukemia, droplet digital PCR, IGHV mutational status, molecular monitoring

## Abstract

In chronic lymphocytic leukemia (CLL), *NOTCH1* gene mutations (*NOTCH1*^mut^) have been associated with adverse prognostic features but the independence of these as a prognostic factor is still controversial. In our study we validated a c.7541-7542delCT *NOTCH1* mutation assay based on droplet digital PCR (ddPCR); we also analyzed the *NOTCH1*^mut^ allelic burden, expressed as fractional abundance (FA), in 88 CLL patients at diagnosis to assess its prognostic role and made a longitudinal ddPCR analysis in 10 cases harboring *NOTCH1*^mut^ to verify the FA variation over time. Our data revealed that with the ddPCR approach the incidence of *NOTCH1*^mut^ in CLL was much higher (53.4%) than expected. However, longitudinal ddPCR analysis of CLL cases showed a statistically significant reduction of the *NOTCH1*^mut^ FA detected at diagnosis after treatment (median FA 11.67 % vs 0.09 %, respectively, p = 0.01); the same difference, in terms of *NOTCH1*^mut^ FA, was observed in the relapsed cases compared to the *NOTCH1*^mut^ allelic fraction observed in patients in complete or partial remission (median FA 4.75% vs 0.43%, respectively, p = 0.007). Our study demonstrated a much higher incidence of *NOTCH1*^mut^ in CLL than has previously been reported, and showed that the *NOTCH1*^mut^ allelic burden evaluation by ddPCR might identify patients in need of a closer clinical follow-up during the “watch and wait” interval and after standard chemotherapy.

## INTRODUCTION

Chronic lymphocytic leukemia (CLL) is a heterogeneous disease with highly variable clinical manifestations, ranging from asymptomatic at the time of diagnosis to a progressive symptomatic disease that is poorly responsive to the common immuno-chemotherapeutic regimens [1–2]. Genomic sequencing studies have revealed a number of recurrently mutated genes in CLL [3–5]. Among them, mutations of the *NOTCH1* gene are found at diagnosis with a variable incidence depending on the method used, ranging from 7% to 22% of CLL cases [6–10], and up to 20-30% in chemo-refractory disease and cases transformed to the Richter syndrome, respectively [11–14]. Moreover, a greater burden of *NOTCH1* mutations (*NOTCH1*^mut^) has been reported in CLL patient subgroups defined by trisomy 12 and an unmutated *IGHV* gene status [8, 15–16]. Some studies have suggested that *NOTCH1* mutated cases exhibit adverse prognostic features with a shorter time to first treatment (TTT) and overall survival (OS), but this point is still controversial [7–10, 17–18]. A two base-pair frameshift-deletion (c.7544_7545fsdelCT) accounts for more than 90% of all *NOTCH1*^mut^ in CLL and results in a stable activated form of *NOTCH1* due to the truncation of the C-terminal PEST domain, which is involved in *NOTCH1* protein turnover and degradation [4]. Apart from next generation (NGS) and Sanger sequencing analysis, the presence of c.7541-7542delCT *NOTCH1* mutations (*NOTCH1*^mut^) can be investigated by amplification refractory mutation system PCR, as previously reported [6, 9, 13, 15]. Obviously, all of these methods of investigation, with the exception of NGS, provide only qualitative and not quantitative information. We validated a c.7541-7542delCT *NOTCH1* specific mutation assay based on digital droplet PCR (ddPCR) technology. This technique relies on the fragmentation of the PCR reactions into very small droplets that are clonally analyzed [19]. The purpose of ddPCR is to quantify the absolute number of target present in a sample, implementing PCR data with Poisson statistics [20]. Therefore, the ddPCR provides a more direct measurement of target genomic copy numbers and offers a greater precision and reproducibility. The aims of our study were to analyze: a) a c.7541-7542delCT *NOTCH1* mutation assay based on ddPCR; b) the *NOTCH1*^mut^ allelic burden in CLL patients at diagnosis to assess its prognostic role; c) longitudinal ddPCR in a few cases harboring *NOTCH1*^mut^ (including patients who required therapy and those who were managed with a “watch and wait” approach).

## RESULTS

**Figure 1:**
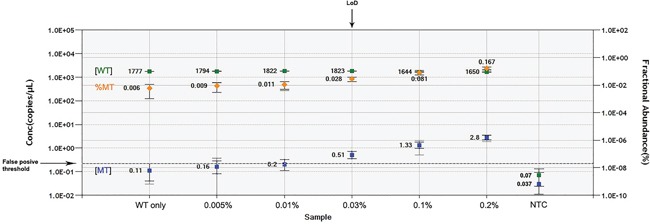
LOD determination Wild-type (WT) template concentration in copies per μL (cpm) (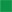), fractional abundance of mutant to WT template (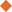) and mutant template cpm (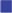) are reported for each dilution of pSC/*NOTCH1*_mut on the genomic WT background. The *False positive threshold* (dashed line) was determined as the upper limit of the mutant concentration error bars of the “WT only” control. The value of *LOD* was equal to the dilution value where the cpm of mutated target fell just above the threshold. NTC, no template control.

**Figure 2:**
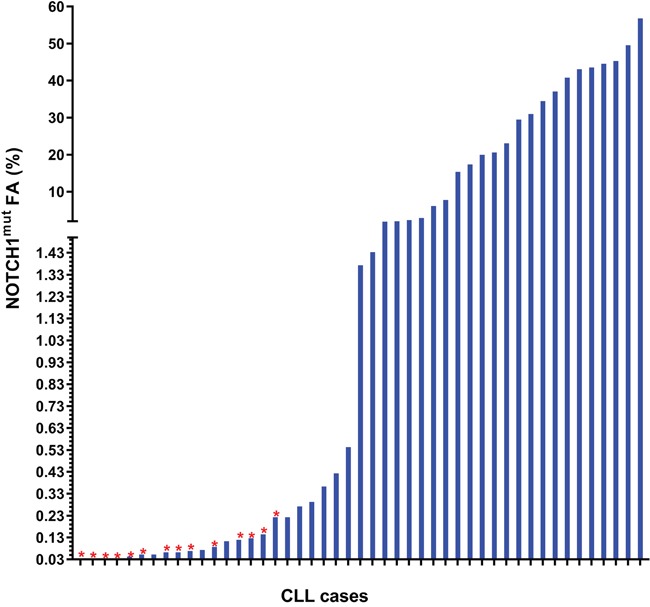
Frequency distribution of the *NOTCH1*^mut^ FA in CLL patients resulting positive for mutations at ddPCR The red star (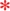) shows the cases resulting negative for NOTCH1^mut^ by AS-PCR.

**Figure 3:**
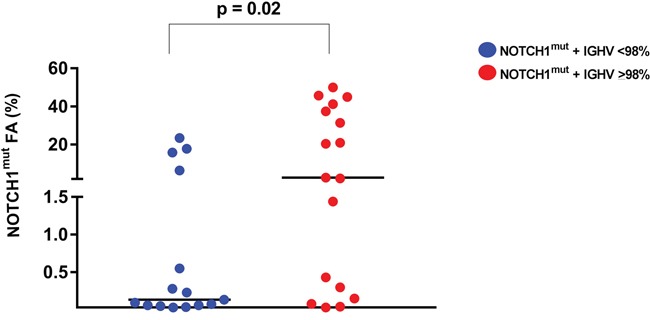
*NOTCH1*^mut^ allelic burden in CLL patients The allelic fraction of *NOTCH1*^mut^ detected by ddPCR resulted higher in CLL patients with unmutated *IGHV*. Each dot represents a patient. The lines indicate the median for each group.

**Figure 4:**
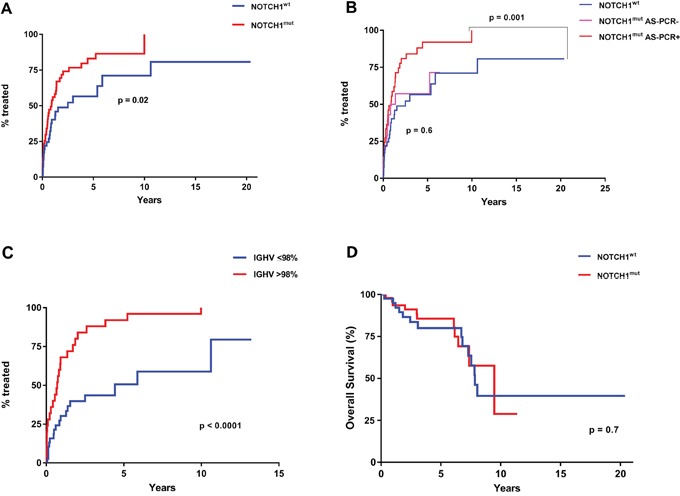
TTT and OS according to *NOTCH1*^mut^ and *IGHV* mutational status **A**. Comparison of TTT among patients carrying *NOTCH1*^mut^ (red line) and those with *NOTCH1*^wt^ (blue line) (p= 0.02 for *NOTCH1*^mut^ vs *NOTCH1*^wt^) **B**. Comparison of TTT among CLL cases resulting positive for the detection of *NOTCH1*^mut^ (red line) at ddPCR and AS-PCR, those positive at ddPCR and negative at AS-PCR (pink line), and cases carrying a *NOTCH1*^wt^ gene (blue line) (p = 0.001 for *NOTCH1*^mut^ cases detected by ddPCR and AS-PCR vs *NOTCH1*^wt^; p = 0.6 for *NOTCH1*^mut^ detected by ddPCR but negative for AS-PCR vs *NOTCH1*^wt^. **C**. Comparison of TTT among patients carrying the mutated (blue line) or unmutated *IGHV* gene sequence (red line) (p < 0.0001). **D**. Comparison of OS among patients carrying *NOTCH1*^mut^ (red line) and those with *NOTCH1*^wt^ (blue line) (p= 0.7)

**Figure 5:**
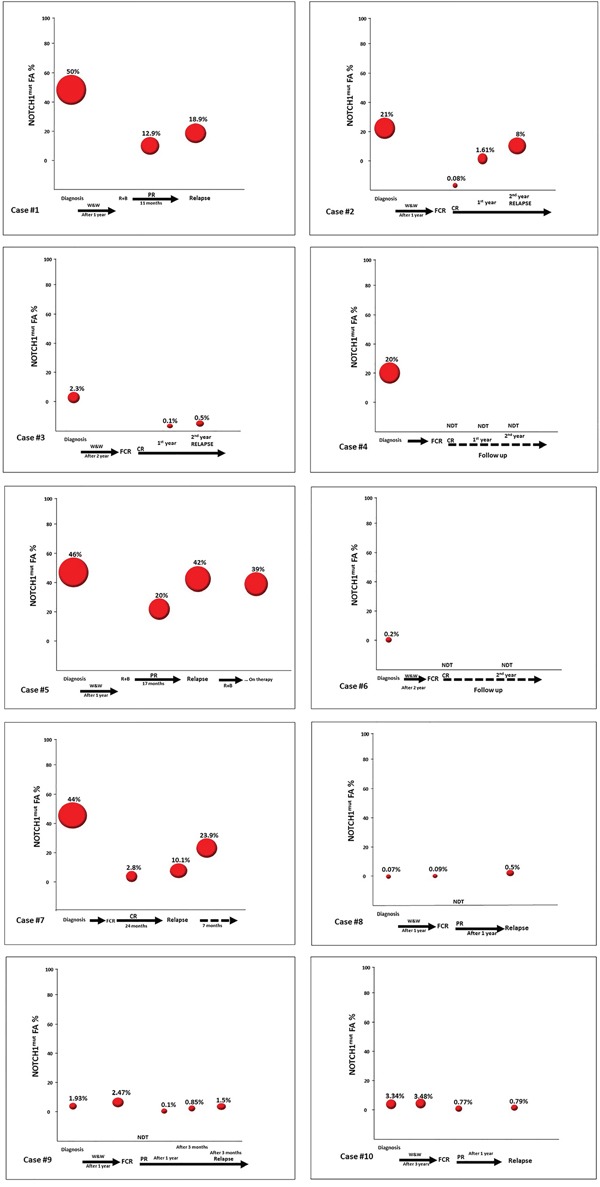
Longitudinal analysis of CLL patients harboring *NOTCH1*^mut^ **A**. The line graph represents the *NOTCH1*^mut^ allelic burden modification for each case (10 patients) before (at diagnosis) and post treatment. **B**. The line graph represents the *NOTCH1*^mut^ allelic burden modification for each case (8 patients) at the time of CR/PR and at CLL relapse.

**Figure 6:**
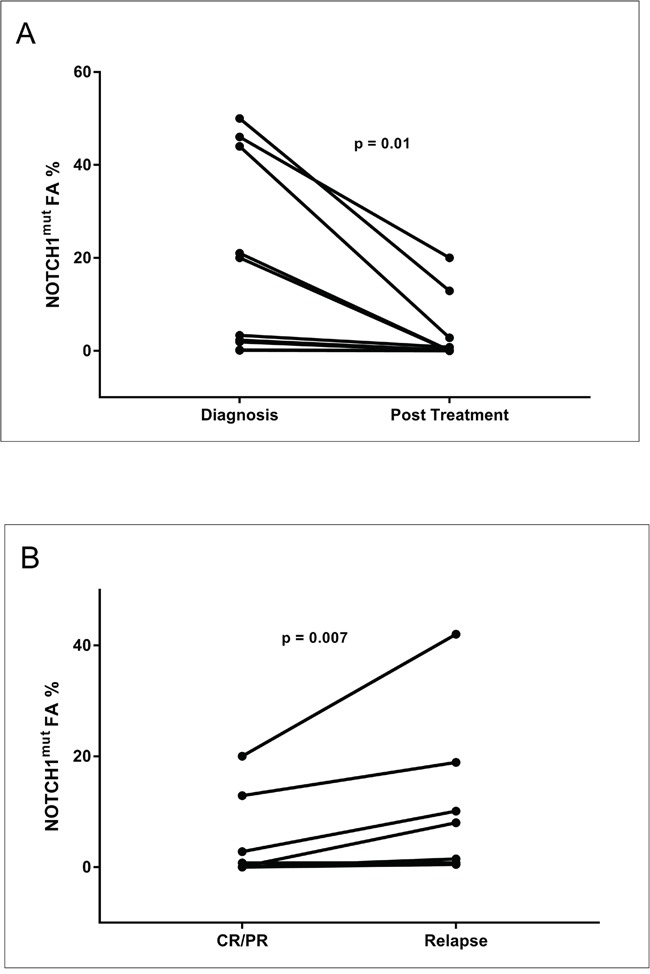
Longitudinal analysis of the *NOTCH1*^mut^ FA variations in CLL patients Graphical illustration of the kinetics of the *NOTCH1* mut clone in 10 CLL patients longitudinally investigated by ddPCR. All patients were treated; all relapsed except Cases #4 and #6. W&W, watch and wait; FCR, fludarabine, cyclophosphamide, rituximab; BR, bendamustine, rituximab; CR, complete response according to the IWCLL-NCI criteria; PR, partial response according to IWCLL-NCI criteria.

The AS-PCR approach demonstrated *NOTCH1*^mut^ in a total of 33/88 (37.5%) CLL patients. ddPCR experiments aimed at detecting the LoD for the *NOTCH1*^mut^ assay established that the limit was a FA ≥0.03% (Figure 1), that is, it can detect three positive events of a total of 10.000 events. A higher percentage of CLL patients bearing *NOTCH1*^mut^ was detected by ddPCR analysis, 47/88 (53.4%) patients resulting positive, with a FA ≥ 0.03% (Figure 2). In particular, among the 14 patients positive at ddPCR but negative at AS-PCR (*NOTCH1*^mut^/AS-PCR-) the median FA was 0.07% (min. 0.03% – max 0.23%) (Figure 2). Among all cases with *NOTCH1*^mut^, 16 (18.1%) had a FA >15%, a value roughly within the detection limit of Sanger sequencing. The *IGHV* status was assessed in 63 (71.5%) cases, *NOTCH1*^mut^ mostly occurred in *IGHV*-unmutated CLL patients (15/32 (46.8%) vs 8/31 (25.8%) in *NOTCH1*^wt^ patients, p = 0.03). Interestingly, among all the CLL cases bearing *NOTCH1*^mut^ those with unmutated *IGHV* had a higher median FA than the value observed in *IGHV*-mutated patients (2.7% vs 0.1%, respectively, p = 0.02) (Figure 3). We did not find statistically significant associations between *NOTCH1* mutational status and the clinical and biological features of our CLL series, such as age, sex, stage, and FISH abnormalities (Table 1). *NOTCH1*^mut^ had a significant effect on TTT; in fact, the median TTT was shorter in the *NOTCH1*^mut^ group compared to *NOTCH1*^wt^ patients (0.9 vs 2.5 years, respectively, p = 0.02) (Figure 4A). Moreover, considering in TTT analysis also the *NOTCH1*^mut^/AS-PCR- subgroup, the latter did not show statistically significant differences from *NOTCH1*^wt^ patients (Figure 4B). At univariate analysis the other covariate with a significant impact on TTT was *IGHV* mutational status: *IGHV*-mutated patients had a longer TTT than the *IGHV*-unmutated group (4.43 vs 0.71, p<0.0001) (Figure 4C). Moreover, in a four variables multivariate analysis model that included Rai stage, *NOTCH1*^mut^, and FISH abnormalities, *IGHV* mutational status emerged as the sole independent prognostic factor for TTT (hazard ratio = 3.16; 95% confidence interval = 1.54-6.45; p = 0.001). There was no statistically significant difference in the median OS between patients harboring *NOTCH1^mut^* and those with the wild-type gene (9.4 vs 7.8 years, respectively, p = 0.7) (Figure 4D); in our series no other covariates (stage, FISH abnormalities, and *IGHV* status) were able to predict a better OS (data not shown). Longitudinal ddPCR analysis of sequential samples of CLL patients bearing *NOTCH1*^mut^ was carried out in: a) 5 cases harboring unmutated *IGHV* (Cases #1 - #5); b) 1 case with *IGHV* mutated status (Case #6); c) 3 cases bearing a not productive *IGHV* rearrangement (Cases #7- #9); d) 1 case with no tested *IGHV* mutational status (Case #10) (Figure 5). All cases were treated and showed a statistically significant reduction of the *NOTCH1*^mut^ allelic burden detected at diagnosis after treatment (median FA 11.67 % vs 0.09 %, p = 0.01) (Figure 6A). Among these cases, eight relapsed after the treatment; in these cases, at the time of the CLL relapse the *NOTCH1*^mut^ allelic fraction was higher compared with the value observed in complete (CR) or partial remission (PR) (median FA 4.75% vs 0.43%, respectively, p = 0.007) (Figure 6B). In two cases in which CLL relapse did not occur (Cases #4 and #6) *NOTCH1*^mut^ became undetectable by ddPCR analysis during follow-up.

**Table 1:** Main features of CLL patients

	*NOTCH1*^wt^ (n = 41)n(%)	*NOTCH1*^mut^ (n = 47)n(%)	p value
Age, years: median (range)	57 (40-80)	62 (40-82)	0.1
Sex, Male/Female	27 (66%)/14 (34%)	34 (72%)/13 (28%)	0.6
Rai stage 0-1	24 (58%)	24 (51%)	0.5
IGHV homology ≥ 98%	8 (25.8%)	15 (46.8%)	0.03
IGHV homology < 98%	23 (74.2%)	17 (53.2%)	
FISH abnormalities	23 (74%)	33 (80%)	0.5
Normal FISH	8 (26%)	8 (20%)	
del13q14 as unique lesion	8 (26%)	10 (24%)	0.7
No del13q14 as unique lesion	15 (65%)	23 (70%)	
+12	7 (31%)	14 (34%)	0.3
+12 neg	24 (69%)	27 (66%)	
del11q23	3 (9.6%)	8 (19.5%)	0.3
del11q23 neg	28 (90.4%)	33 (79.5%)	
del17p13	6 (19.3%)	5 (12.1%)	0.5
del17p13 neg	25 (80.7%)	36 (87.9%)	
Unfavorable aberrations(del11q23 or del17p13)	9 (29%)	12 (29.2%)	0.9
No unfavourable aberrations	22 (71%)	29 (70.8%)	
AS-PCR +/-	0 / 31 (100%)	33 (70.2%)/14 (29.8%)	
Treated patients	24 (59%)	39 (83%)	0.2
TTT (years)	2.5	0.9	0.02
OS (years)	7.84	9.46	0.2

## DISCUSSION

The ddPCR technology is a third-generation PCR method that is being applied to various fields of medical diagnostics with promising results. ddPCR is easy to perform and does not require replicate analysis or the generation of standard curves for target quantification. In fact, in comparison with quantitative analog PCR, ddPCR has the potential to make quantitative analyses more reproducible, and precise quantitation might facilitate a variety of clinical tests. However, there are only few studies focused on the use of ddPCR in hematological malignancies [21–26]. In a recent report, *NOTCH1*^mut^ were detected by NGS in 11% of monoclonal B cell lymphocytosis (MBL) and 13.4% of CLL Binet stage A patients [27]; moreover, this mutation was frequently observed at a low clonal level, particularly in MBL patients, and sequential analyses demonstrated that the *NOTCH1*^mut^ generally did not appear during the disease course, and that the mutational burden in positive cases remained stable over time. Moreover, it has also been reported that among untreated early stage CLL patients the incidence of *NOTCH1*^mut^ did not appear to increase over time [27–29]. These data are consistent with the view that *NOTCH1*^mut^ are an early event, and that a fraction of *NOTCH1* mutations may be restricted to small subclones that are not detectable by conventional sequencing at the time of CLL presentation. In our study, we employed a ddPCR assay which allows easy measurement of the absolute copies number of the *NOTCH1*^mut^ allele. The increased sensitivity of our ddPCR assay revealed that the incidence of *NOTCH1*^mut^ in CLL patients was much higher than expected. On the other hand, considering only the cases bearing a mutated allelic fraction value within the Sanger's detection limit (10-20%) the frequency of *NOTCH1*^mut^ in our series was in accordance with the previously reported values. As in other studies, our data confirmed the association of *NOTCH1*^mut^ with *IGHV*-unmutated CLL [6, 15, 23–24]; moreover, our finding that among all the CLL patients with *NOTCH1*^mut^ those with *IGHV*-unmutated had the higher mutated allelic burden seems worthy of note. This could mean that the prognostic potential of the *NOTCH1*^mut^ may be intrinsically correlated with several biologic factors, other than *IGHV* mutational status, pertaining to the CLL patient. Although in our series the presence of *NOTCH1*^mut^ was associated with a shorter TTT, this finding was not confirmed at multivariate analysis, where the *IGHV* mutational status appeared as the sole independent prognostic factor. This result was in accordance with some previous reports [8, 10, 18] but in contrast with others [7, 9, 17]. The discrepancy concerning the prognostic role of *NOTCH1*^mut^ appears more comprehensible in light of our findings, that unveil the high incidence of *NOTCH1*^mut^ associated with an extreme heterogeneity in CLL patients, in terms of the mutated allelic burden. In fact, when we considered, among all the *NOTCH1*^mut^ cases, those resulting negative for mutations at AS-PCR as a distinct subgroup, there was no difference in terms of TTT compared to the *NOTCH1*^wt^ patients group. Therefore, it seems reasonable to assume that the amount of mutated gene, rather than the mere presence of the mutation, associated with other factors (such as the *IGHV* mutational status) better defines the prognostic role of *NOTCH1*^mut^ in CLL patients. Further data will be needed to support this hypothesis.

The sequential analysis results suggest some observations. Firstly, all cases with *NOTCH1*^mut^ showed a decrease of the mutated allelic burden at the end of the induction treatment, when achieving a CR or PR; this fact appears very interesting because it suggests that *NOTCH1*^mut^ may be a possible molecular marker of response to therapy, that is very easy to measure with the ddPCR approach. Secondly, in three cases (Cases #8, #9, and #10) (Figure 5) the *NOTCH1*^mut^ allelic burden increased over time, from “watch and wait” up to the standard chemotherapy approach; in this context it is possible that ddPCR may be a useful tool in CLL patients candidates for the “watch and wait” strategy molecular monitoring, and more predictive than the classic clinical/laboratory parameters. Obviously, this latter consideration appears speculative and needs additional data to support such a use. Finally, all relapsed cases showed a *NOTCH1*^mut^ allelic burden increase at the time of relapse. This finding further supports the hypothesis that monitoring *NOTCH1*^mut^ by ddPCR could be employed as a marker of minimal residual disease. Moreover, it is noteworthy that in the two cases characterized by a continuous CR (Cases #4, #6) (Figure 5) the *NOTCH1*^mut^ has remained undetectable during follow-up.

In conclusion, our ddPCR assay is a valid tool, and its high sensitivity revealed a much higher incidence of *NOTCH1*^mut^ in CLL patients than expected. The *NOTCH1*^mut^ prognostic significance remains to be fully clarified. Our findings suggest that *NOTCH1*^mut^ allelic burden evaluation by ddPCR might identify patients in need of closer clinical follow-up during the “watch and wait” interval and after the standard chemotherapy approach.

## MATERIALS AND METHODS

### Patients and samples

Samples from 88 CLL patients at diagnosis were included in this study. All patients were diagnosed according to NCI criteria [30]. Table 1 summarizes the clinicobiologic characteristics of the cohort. The median age of the entire series was 60 years (range, 40–82 years); there were 61 males and 27 females. Median follow-up was 3.9 years. In 10 patients, longitudinal samples obtained at different CLL time-points (progression before treatment, after chemotherapy, at relapse) were also examined. The study was approved by the local ethics committee. All patients gave informed consent to participate in this study.

### Molecular analysis

Genomic DNA of CLL patients was extracted from peripheral blood at diagnosis using the QIAamp DNA Blood Mini Kit (Qiagen), and quantified with a Qubit 2.0 Fluorometer (Life Technologies). At both CLL diagnosis and relapse, the fraction of tumor cells (CD5^+^CD19^+^) corresponded to 70%-98% as assessed by flow cytometry.

### Sequence analysis of *IGHV* –IGHD–IGHJ rearrangements

PCR amplification and sequence analysis of *IGHV*–*IGHD–IGHJ* rearrangements were performed in 63 (71.5%) cases, as previously described [31].

### AS-PCR

*NOTCH1*^mut^ was investigated by allele-specific PCR (AS-PCR), as previously described [9]; the lower detection limit of this assay was established as 0.1% of the mutant allele burden in serial dilutions of DNA from a *NOTCH1*^mut^ heterozygous sample and a *NOTCH1* wild-type (WT) sample.

### ddPCR mutation detection assay

ddPCR analysis performed by the QX200 (BioRad) system combines water-oil emulsion droplet technology with microfluidics. Each sample is partitioned into 20,000 droplets by a droplet generator and each droplet is amplified by PCR. Then, droplets are analyzed by a droplet reader, which counts the positive and negative fluorescent droplets to define the target concentration. The *NOTCH1*^mut^ detection by ddPCR was conducted using the specific PrimePCR ddPCR Mutation Assays dHsaCP2500501 and dHsaCP2500500 (BioRad) according to the manufacturing protocol. ddPCR was performed by adding 5U of restriction enzyme HAE III (New England Biolabs) with 130ng of DNA template in a final volume of 20μl. After amplification, the 96-well PCR plate was loaded on the Bio-Rad QX200 droplet reader and ddPCR data were analyzed with QuantaSoft analysis software (version 1.7.4). The latter measures the number of positive and negative droplets for each probe (*NOTCH1*^mut^ and *NOTCH1*^wt^) in each sample and calculates the fraction of positive droplets by a Poisson algorithm to determine the concentration of the target. Then, the software returns those data as the fractional abundance (FA) of mutant to wild type template. The FA is calculated as the percentage ratio between the number of mutant DNA molecules (a) and the number of mutant (a) plus wild type (b) molecules (fractional abundance: (a/a+b)).

### Cloning of *NOTCH1* mutant fragment

Genomic DNA isolated from a *NOTCH1*^mut^ patient was amplified with the primers: NOTCHesF (5’-CAGCCAGCAAACATCCAGC-3’) and NOTCHesR (5’-AAAAGGCTCCTCTGGTCGG-3’). The PCR reaction was performed in a final volume of 25μl, containing 100ng of genomic DNA, 1X PCR Buffer, 1.6mM MgCl2, 0.2mM of each dNTP, 0.2μM of NOTCHesF primer, 0.2μM of NOTCHesR primer and 0.5U of Taq DNA Polymerase Recombinant (Invitrogen). Thermal conditions were : 94°C for 3’ (one cycle); 94°C for 30’’, 57°C for 30’’, 72°C for 30’’ (34 cycles); and final elongation at 72°C for 5’. Products were resolved on 1.5% agarose gels by electrophoresis. The product of 571bp was gel purified (QIAquick Gel Extraction Kit QIAGEN) prior to performing the cloning reaction. Cloning was conducted with the StrataClone PCR Cloning Kit (Agilent Technologies) according to the manufacturing protocol.

The positive white colonies were further screened by EcoR I (New England Biolabs) plasmid digestion to verify the presence of the 571bp insert, and by specific *NOTCH1* AS-PCR to detect the mutation. Mutation carrying clones were also confirmed by Sanger sequencing and one was selected as the mutant template for LoD determination (named pSC/*NOTCH1*_mut).

### ddPCR assay limit of detection

The limit of detection (LOD) is defined as the lowest mutant concentration that can be reliably distinguished from the WT only control. For determination of the LOD it is necessary to run a plate containing no template control (NTC) wells, WT only control wells and serial dilutions of positive control mutant template in a constant background of WT DNA. We prepared a serial dilution of pSC/*NOTCH1*_mut (0.005% - 0.2%) on a genomic WT background (2.000 copies per microliter about 130ng).

After ddPCR detection, the FA and concentration were calculated and plotted. The false positive threshold of the assay was determined as the upper limit of the mutant concentration error bars in the WT-only. LoD was calculated as the lowest mutant concentration where the lower limit of the error bars does not cross the false positive threshold. The first concentration value above the threshold represents the lowest statistically significant (p<0.05) detectable concentration.

### Fluorescence in situ hybridization (FISH)

FISH analyses were performed using bacterial artificial chromosomes (Children's Hospital Oakland Research Institute, Oakland, CA, USA), specific for 17p13, 11q22, 12q and 13q14 chromosomes (RP11-199F11, RP11-835M17, RP11-1100L3, and RP11-153K13, respectively) according to the University of California (Santa Cruz, CA, USA) database (http://genome.ucsc.edu/ February 2009 release). Chromosome preparations were hybridized in situ with probes labeled by nick translation, as previously described [32–33].

### Statistical analysis

Clinical and biological features between groups were compared using the Fisher exact test for categorical data and the nonparametric Mann-Whitney U or t tests for continuous variables; continuous variables from related samples were compared using the Wilcoxon test. A p value <0.05 was considered significant. OS was calculated from the date of diagnosis to the date of death or last follow-up. TTT was calculated from the date of diagnosis to the date of first treatment or last follow-up, considering disease-unrelated deaths as competing events. The log-rank test was used to compare Kaplan-Meier curves of OS; the Gray test was used to compare cumulative incidence curves of TTT. Multivariate analyses of prognostic factors were modeled using Cox and Fine-Gray regression models as previously described [34]. Statistical analyses were carried out using GraphPad Prism version 7.0 for Windows (GraphPad Software, San Diego, CA) and XLSTAT version 2014. 5.03 (AddinsoftTM).
